# Characteristics of Preteen Suicide in Japan

**DOI:** 10.1001/jamanetworkopen.2024.55471

**Published:** 2025-01-22

**Authors:** Yuka Nishina, Yusuke Yagai, Rinko Goto, Mayumi Hangai

**Affiliations:** 1 Japan Suicide Countermeasures Promotion Center, Tokyo, Japan

## Abstract

This cross-sectional study evaluates characteristics of preteen suicide rates in Japan over the past 15 years using data from national suicide statistics.

## Introduction

Youth suicides have become a global concern in recent years. Although Japan’s overall suicide rate has declined after the enactment of the Basic Act on Suicide Countermeasures in 2006, suicide remains the leading cause of death among teenagers.^1^ A report from the US indicates an increase in suicide rates among preteens aged 8 to 12 years.^2^ However, no similar analysis has been conducted in Japan for this age group that we know of. This study aims to fill this gap by analyzing the trends and characteristics of preteen suicide rates in Japan over the past 15 years using data from national suicide statistics.

## Methods

This cross-sectional study received approval from the research ethics committee of the Japan Suicide Countermeasures Promotion Center and followed the Strengthening the Reporting of Observational Studies in Epidemiology (STROBE) reporting guidelines. The need for informed consent was waived because data were deidentified. The study relied on data from the National Police Agency’s suicide statistics and population estimates from the Ministry of Internal Affairs and Communications, covering 2009 to 2023.^3^ We extracted information on suicide cases involving individuals aged 8 to 12 years and analyzed the temporal changes in suicide mortality rates, including the annual percentage change (APC), which shows the increase in rate from the previous year. The study duration was divided into 2 periods (January 1, 2009 to December 31, 2015 and January 1, 2016 to December 31, 2023) to compare suicide mortality rates and case attributes between these time frames. The attributes, including sex, age, method of suicide, region, and history of suicide attempts were obtained from the suicide statistics. No variables were adjusted. Incidence rate ratios (IRRs) of the suicide rate for the latter half compared with the first period, along with 95% CIs and APC, were calculated using R version 4.3.2 (R Project for Statistical Computing). CIs excluding 1.00 were considered statistically significant.

## Results

Between 2009 and 2023 in Japan, 283 young individuals aged 8 to 12 years died by suicide (159 male individuals [56.2%]; 124 female individuals [43.8%]). The overall suicide rate showed an upward trend (APC, 16.15%; 95% CI, –9.15% to 41.45%) with a greater increase among female individuals (APC, 38.44%; 95% CI, −14.66% to 91.55%) (Figure). The study identified a significant escalation in suicide rates between 2009 to 2015 and 2016 to 2023, rising from 2.84 to 4.03 per 1 million (IRR, 1.42; 95% CI, 1.12 to 1.80). This increase was particularly pronounced among female individuals (IRR, 1.98; 95% CI, 1.36 to 2.88) and those aged 12 years (IRR, 1.67; 95% CI, 1.24 to 2.27), and those using lethal methods including jumping from heights (IRR 2.62; 95% CI, 1.53 to 4.49). Regionally, western Japan and nonmetropolitan areas experienced the most significant increases (IRR, 1.93; 95% CI, 1.25 to 2.99 and IRR, 1.43; 95% CI, 1.07 to 1.90, respectively). Additionally, a significant increase was observed in children with a history of suicide attempts, as well as from April to June in the latter period (2016 to 2023) (Table).

**Figure.**
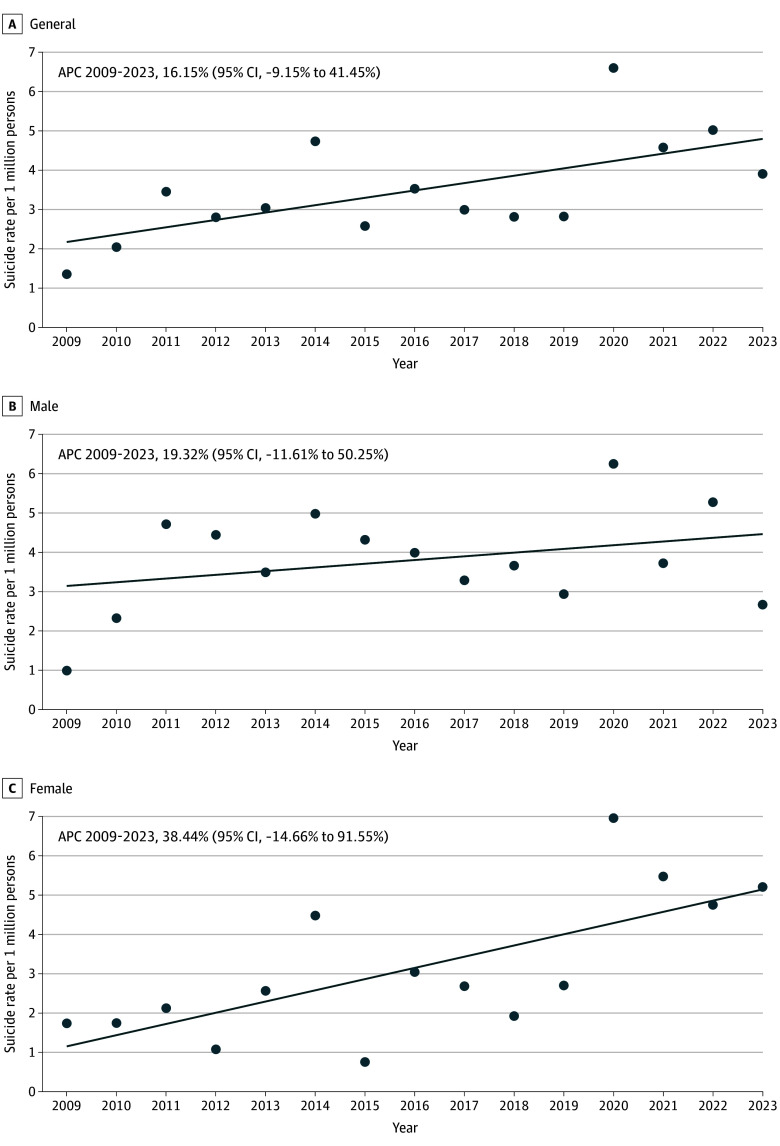
Trends in Suicide Rates for Youths Aged 8 to 12 Years in Japan Between 2009 and 2023 Crude rates per 1 million persons were calculated using suicide statistics and population estimates from the National Police Agency and the Ministry of Internal Affairs and Communications, respectively. Data markers indicate observed rates; suicide rate trends are displayed as solid-colored lines. The increase rate from the previous year was calculated using the annual suicide mortality rates as follows: increase rate = ([suicide mortality rate of the current year / suicide mortality rate of the previous year] – 1) × 100. APC and standard error were obtained, and based on this standard error, a 95% CI was calculated. APC indicates annual percentage change.

**Table.** Period Trends in Suicide Rates for Preteens in Japan

Characteristic	No. of persons (rate per 1 million persons)		Period trend, 2009-2015 to 2016-2023, IRR (95% CI)^c^
	2009-2015^a^	2016-2023^b^	
Overall	113 (2.84)	170 (4.03)	1.42 (1.12-1.80)^d^
Sex			
Male	73 (3.58)	86 (3.98)	1.11 (0.81-1.52)
Female	40 (2.06)	84 (4.08)	1.98 (1.36-2.88)^d^
Age, y			
8-9	9 (0.58)	4 (0.24)	0.41 (0.13-1.35)
10	6 (0.75)	9 (1.06)	1.41 (0.50-3.97)
11	32 (3.96)	41 (4.82)	1.22 (0.77-1.03)
12	66 (8.07)	116 (13.51)	1.67 (1.24-2.27)^d^
Suicide method			
Hanging	89 (2.24)	112 (2.65)	1.19 (0.90-1.57)
Jumping from heights	18 (0.45)	50 (1.18)	2.62 (1.53-4.49)^d^
Transportation-related	4 (0.10)	7 (0.17)	1.65 (0.48-5.63)
Others	2 (0.05)	1 (0.02)	NC^e^
Region			
North	16 (3.65)	20 (4.55)	1.25 (0.65-2.41)
East	67 (3.30)	88 (3.99)	1.21 (0.88-1.66)
West	30 (2.00)	62 (3.87)	1.93 (1.25-2.99)^d^
Area of residence			
Metropolitan^f^	35 (3.38)	53 (4.51)	1.34 (0.87-2.05)
Nonmetropolitan	78 (2.66)	117 (3.81)	1.43 (1.07-1.90)^d^
History of suicide attempts			
Yes	6 (0.15)	19 (0.45)	2.98 (1.19-7.47)^d^
No	95 (2.39)	137 (3.25)	1.36 (1.05-1.77)^d^
Unknown	12 (0.30)	14 (0.33)	1.10 (0.51-2.38)
Quarter			
January-March	24 (0.60)	32 (0.76)	1.26 (0.74-2.13)
April-June	20 (0.50)	41 (0.97)	1.93 (1.13-3.30)^d^
July-September	40 (1.01)	58 (1.37)	1.37 (0.91-2.04)
October-December	29 (0.73)	39 (0.92)	1.27 (0.78-2.05)

Abbreviations: IRR, incidence rate ratio; NC, not calculated.

^a^ From January 1, 2009, to December 31, 2015.

^b^ From January 1, 2016, to December 31, 2023.

^c^ The period analyzed is from January 1, 2015, to December 31, 2023. This term is divided into 2 before and after the midpoint. IRR was calculated as (suicide rate in 2016 – 2023) / (suicide rate in 2019 – 2015).

^d^ Confidence intervals that excluded 1.00 were considered statistically significant.

^e^ Not calculated because the number of deaths was extremely small.

^f^ Refers to the major metropolitan area in Japan, which includes the 23 special wards of Tokyo prefecture and the ordinance-designated cities with a population of 500 000 or more.

## Discussion

This study found significant increases in suicide rates among preteens in Japan between the periods 2009 to 2015 and 2016 to 2023, mirroring the trends observed in the US.^2^ The increase was more pronounced among female preteens, which differs from the general trend of higher suicide rates among males.^1^ While jumping from heights accounts for approximately 12% of suicides in the general population,^4^ this age group exhibited a higher proportion. The increase in jumping among this age group is possibly indicative of more impulsive decisions, as such methods typically require minimal preparation compared with others.^5^ The stress associated with the start of the school year in April may have increased in recent years, as reflected by the rise in second-quarter suicide rates between 2016 to 2023 compared with 2009 to 2015. The rise in suicides among children with a history of suicide attempts highlights the need for targeted interventions for survivors, given that prior attempts are a major risk factor.^6^ The suicide statistics are based on police investigations and interviews with bereaved families, which may lead to potential misclassification, particularly regarding the history of suicide attempts, as stigma around suicide might influence reporting. Due to the limited data, interaction is not assessed. Despite these limitations, the findings emphasize the necessity of strengthening support for at-risk children. This study was conducted among preteens in Japan; however, the findings suggest the need to observe similar trends across other age groups and countries in future research.

## Notes

Supplement.
Data Sharing Statement
