# Supplementary material for: Characteristics of Preteen Suicide in Japan
Source: JAMA Netw Open. 2025 Jan 22;8(1):e2455471. doi: 10.1001/jamanetworkopen.2024.55471 (PMC11755189; doi:10.1001/jamanetworkopen.2024.55471)
Supplement: Supplement. — Data Sharing Statement [file jamanetwopen-e2455471-s001.pdf]

Data Sharing Statement

Nishina. Trends and Characteristics of Preteen Suicide in Japan. *JAMA Netw Open*. Published January 22, 2025. doi:10.1001/jamanetworkopen.2024.55471

Data
Data available: No

Additional Information
Explanation for why data not available: The data analyzed was provided by the National Police Agency through a special procedure. However, as the agency has not made this data publicly available, it cannot be disclosed.
